# Evaluation of liver fibrosis in patients with Wilson’s disease

**DOI:** 10.1097/MEG.0000000000001754

**Published:** 2020-05-15

**Authors:** Adam Przybyłkowski, Jowita Szeligowska, Magdalena Januszewicz, Joanna Raszeja-Wyszomirska, Benedykt Szczepankiewicz, Piotr Nehring, Barbara Górnicka, Tomasz Litwin, Anna Członkowska

**Affiliations:** a Department of Gastroenterology and Internal Medicine; b Second Department of Clinical Radiology; c Department of Hepatology and Internal Medicine; d Chair and Department of Pathomorphology, Medical University of Warsaw; e Second Department of Neurology, Institute of Psychiatry and Neurology, Warsaw, Poland

**Keywords:** elastography, liver biopsy, liver stiffness, noninvasive evaluation

## Abstract

**Objectives:**

Staging of fibrosis in chronic liver disease is important for prognosis and treatment planning. Liver biopsy is the gold standard in fibrosis assessment; however, new methods for fibrosis and stiffness measurement exist which have not been evaluated in patients with Wilson’s disease. To evaluate the accuracy of collagen proportionate area (CPA), transient elastography and shear wave elastography (SWE) in the assessment of liver fibrosis in adult patients with Wilson’s disease.

**Methods:**

In this retrospective study of 60 patients with Wilson’s disease, results of percutaneous cutting liver biopsy assessed using the Ishak fibrosis score and CPA were compared with liver stiffness measured with transient elastography and SWE.

**Results:**

CPA correlated with the Ishak score (*r* = 0.45; *P* = 0.001) and transient elastography results correlated with SWE measurements (*r* = 0.80; *P* = 0.0001). In contrast, transient elastography or SWE did not significantly correlate with the Ishak score or CPA.

**Conclusion:**

Collagen content assessment may be useful for estimation of liver fibrosis in patients with Wilson’s disease. However, single time-point elastographic liver stiffness measurements have a limited diagnostic value in Wilson’s disease.

## Introduction

Wilson’s disease is an autosomal recessive inherited disease of impaired excretion and excessive accumulation of copper caused by mutations in the *ATP7B* gene that encodes the ATP7B copper transporter [1]. The worldwide incidence of Wilson’s disease is estimated at 1:30 000 [2]. Accumulation of copper mostly affects the liver, brain and cornea, leading to organ injury. The clinical presentation of liver disease in Wilson’s disease spans asymptomatic elevation of liver enzymes, acute or chronic hepatitis, occult cirrhosis and fulminant hepatic failure [3,4].

Assessment of liver fibrosis in chronic liver diseases has an essential prognostic value. Liver biopsy is the gold standard for diagnosis of liver cirrhosis and fibrosis and several histopathologic scoring systems exist that are based on architectural changes [5]. Classic staining for fibrosis is performed with agents that react uniformly with connective tissue (e.g., reticulin and trichrom) and are not selective for collagen fibers, which may result in inadequate grading of fibrosis. To avoid bias resulting from nonselective staining, Sirius staining may be used that is selective for collagen types 1 and 3 and computer-assisted digital imaging analysis can determine fibrosis density based on the calculation of collagen proportionate area (CPA). However, CPA still requires liver biopsy, which is invasive. In this context, the search for noninvasive, repetitive and reliable methods led to the development of ultrasound-based elastography techniques; the most commonly used of which are transient elastography and shear wave elastography (SWE) [6]. Transient elastography is performed in M-mode without visual control of the ultrasound liver image, while SWE is guided by B-mode imaging. In transient elastography, a mechanical piston placed on the body surface creates shear waves within the liver. The average speed of transmitted shear waves that propagate through the liver is received by pulse-echo ultrasound acquisition. SWE is based on acoustic radiation force impulse technology in which an acoustic beam generates shear waves, which are followed by the ultrasound transducer. In both methods, the speed of shear wave propagation is converted to a tissue elasticity value [6].

Currently, transient elastography and SWE are widely accepted methods for liver fibrosis grading in chronic viral hepatitis B and C, nonalcoholic steatohepatitis and primary sclerosing cholangitis (PSC). Transient elastography and SWE cutoff values for different stages of liver fibrosis are not universal for all liver diseases and must be adjusted for each specific condition.

Both CPA and elastography (transient elastography or SWE) have not been extensively evaluated in Wilson’s disease, and therefore, the aim of this study was to evaluate the accuracy of these methods in the assessment of liver fibrosis in adult patients with Wilson’s disease.

## Patients and methods

### Patients

This was a retrospective study of 60 patients diagnosed with Wilson’s disease, who remained under medical care at the Second Department of Neurology, Institute of Psychiatry and Neurology, Warsaw, Poland, and in the Department of Gastroenterology and Internal Medicine, Medical University of Warsaw, Poland, during the period between 2009 and 2019. Wilson’s disease was diagnosed based on clinical and genetic criteria: serum ceruloplasmin and copper concentration were checked followed by an ocular slit-lamp examination, a 24-h urinary copper excretion assessment and molecular testing for *ATP7B* mutations. The study included patients who had percutaneous liver biopsy, transient elastography and SWE to assess liver fibrosis within a period of six months and who had not been diagnosed with liver cirrhosis previously.

The study was approved by a Bioethics Committee of the Medical University of Warsaw, and all methods were carried out in accordance with relevant guidelines and regulations. As the presented study was retrospective, informed consent was not required.

### Noninvasive liver fibrosis assessment

All patients were fasted before the ultrasound procedure. Liver fibrosis was assessed in all patients by both one-dimensional transient elastography FibroScan (Echosens SA, Paris, France) and two-dimensional SWE integrated in a conventional ultrasound system (Toshiba Aplio 500, Toshiba America Medical Systems, Inc., California, USA) [6]. Liver stiffness was presented in kPa for transient elastography and for SWE [7]. The mean and SD of 10 measurements were calculated and used for further analysis.

Additionally, blood tests were performed to assess levels of aspartate aminotransferase (AST), alanine aminotransferase (ALT), gamma-glutamyltransferase, alkaline phosphatase, total bilirubin, creatinine and albumin and to allow calculation of the international normalized ratio (INR), model of end-stage liver disease (MELD) score, AST-to-platelet ratio index (APRI), FIB-4 score ([age × AST]/[platelet count × √ALT]), Bonacini cirrhosis discriminate score (the sum of scores for platelet count, ALT/AST ratio and INR) and De Ritis ratio (AST/ALT) [8]. Previous recommended cutoff values for liver cirrhosis (APRI ≥ 1, FIB-4 score ≥ 1.45 and ≥ 3.25, Bonacini score > 7 and De Ritis > 1) were used to define positive results; however, these values were based on studies of hepatitis C virus etiology [9–11].

### Standard liver histology

After a period of fasting, percutaneous liver biopsies were performed using a liver cutting Vitesse Biopsy Gun (OptiMed, Ettlingen, Germany), samples were fixed immediately in formalin, prepared in formalin and stained with hematoxylin and eosin. Liver fibrosis was classified according to the Ishak fibrosis score [5,12–14] by two experienced pathologists. The Ishak score classifies liver fibrosis in seven stages (0–7). Stage 0 means no fibrosis, stages 1–3 describe fibrous expansion of some-to-most portal areas with or without fibrous septa with occasional portal to portal bridging in stage 3. Stages 4 and 5 reflect marked bridging with portal-to-portal and/or portal-to-central bridging with occasional nodules in stage 5. Stage 6 describes definite cirrhosis [5]. The grade of inflammation was given as a Knodell Histology Activity Index (HAI) score (minimal for 1–3 points, mild for 4–8 points, moderate for 9–12 points and severe for 13–18 points) [5].

### Collagen proportionate area

Liver samples were stained with PicroSirius red according to the manufacturer’s instructions. Samples were scanned using a Hamamatsu C12000 NanoZoomer-XR Digital Slide Scanner (Hamamatsu, Japan). During image preparation, three representative areas with the most intensive fibrosis assessed microscopically were chosen for each sample (1820 × 1088 pixels) and assessed separately by three researchers. To avoid physiological sequestration of the coloring agent, regions of blood vessels, debris and artifacts were avoided. The percentage of collagen was measured in ImageJ 1.52p (Weyne Resband, National Institutes of Health, Bethesda, Maryland, USA) using the Otsu Threshold method [15,16].

### Statistical analysis

The results were checked for normal distribution. For parametric and nonparametric quantitative variables, Student’s *t* test and Mann–Whitney *U* test were performed, respectively. In qualitative variables analysis, the χ^2^ test was used with Fisher’s correction for small samples if necessary. Missing data were removed in pairs. Statistical significance was recognized when α = 0.05. Multivariate logistic regression was used for regression analysis. Correlations were checked with Spearman’s rank correlation test. Statistical analysis was performed with Statistical data analysis software system (version 13; StatSoft, Poland and Dell Inc., Tulsa, Oklahoma, USA).

## Results

The examined population of 60 patients with Wilson’s disease was equally matched by sex (Table 1). The youngest patient was 18 years old and the oldest was 62 years old, with a mean age of 40 years. All patients were treated with zinc salts or D-penicillamine for at least one year. At biopsy, there was a wide range of symptom duration, ranging from 1 to 49 years, with a median of 8 years.

**Table 1.** Clinical characteristics

	*N* = 60
Sex, *n* (%)	
Females	31 (52)
Males	29 (48)
Age (years), mean ± SD	40.39 ± 12.49
Age at diagnosis (years), mean ± SD	29.28 ± 13.1
Time since Wilson’s disease diagnosis (years), median (IQR)	8 (14)
Laboratory results	
AST (U/l), median (IQR)	30.5 (15)
ALT (U/l), median (IQR)	40 (34.5)
GGT (U/l), median (IQR)	24 (33)
ALP (U/l), median (IQR)	80 (37)
INR, median (IQR)	1.07 (0.10)
Platelets (10^3^/μl), median (IQR)	190 (97)
Bilirubin (mg/dl), median (IQR)	0.63 (0.45)
Creatinine (mg/dl), mean ± SD	0.80 ± 0.16
Albumin (g/l), mean ± SD	4.24 ± 0.43
Noninvasive indirect markers of liver damage	
APRI ≥ 1, *n* (%)	5 (8)
APRI ≥ 0.7, *n* (%)	13 (22)
Bonacini score > 7, *n* (%)	1 (2)
Bonacini score ≥ 5, *n* (%)	30 (50)
De Ritis ratio ≥ 1, *n* (%)	21 (35)
FIB-4 score ≥ 3.25, *n* (%)	34 (57)
FIB-4 score ≥ 1.45, *n* (%)	46 (77)
MELD score, median (IQR)	7 (1)
Liver histology	
Steatosis, *n* (%)	35 (58)
Knodell histology activity index grade	
Minimal, *n* (%	29 (48)
Mild, *n* (%)	30 (50)
Moderate, *n* (%)	1 (2)

Mean ± SD values are presented for variables with normal distribution.

Median (IQR) values are presented for variables with non-normal distribution.

ALP, alkaline phosphatase; ALT, alanine aminotransferase; APRI, AST to platelet ratio index; AST, aspartate aminotransferase; GGT, gamma-glutamyltransferase; INR, International normalized ratio; IQR, interquartile range; MELD, model of end-stage liver disease.

Macrovacuolar steatosis was present in 35 patients (58%; Table 1). A minimal HAI was present in 29 patients (48%), mild in 30 (50%) and moderate in one patient (2%).

Forty-eight patients (80%) had an Ishak score of 3 or less based on liver biopsy, and cirrhosis (Ishak score 5–6) was diagnosed in 5 patients (8%) (Table 2).

**Table 2.** Histological and elastographic findings

		Median (IQR)			
Ishak score	*n*, %	Age, years	Transient elastography, kPa	Shear wave elastography, kPa	Collagen proportionate area, %
1	13 (22)	48 (15.5)	6.6 (3.4)	7.8 (1.9)	3.4 (2.7)
2	13 (22)	39 (17)	7.2 (3.4)	8.3 (1.5)	6.9 (4.3)
3	22 (36)	38.5 (19)	6.2 (3.8)	8.5 (4.1)	5.6 (5.1)
4	7 (12)	43 (31)	5.5 (2.8)	7.9 (2.1)	10.4 (10.3)
5	5 (8)	37.5 (13)	5.0 (3.1)	6.7 (2.1)	12.0 (3.1)

IQR, interquartile range.

**Fig. 1.**
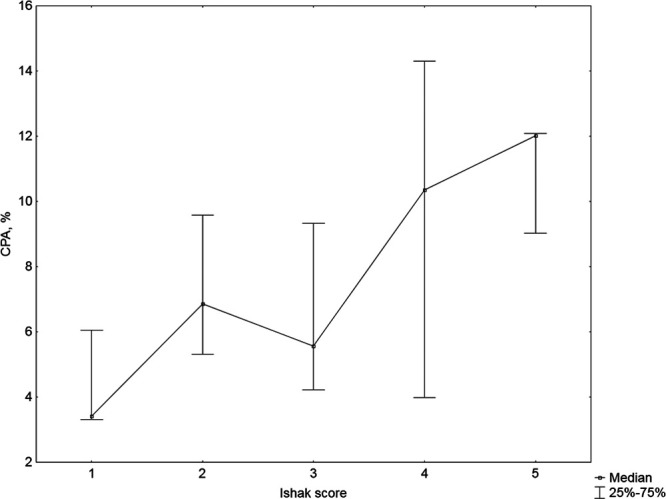
Collagen proportionate area by Ishak score.

**Fig. 2.**
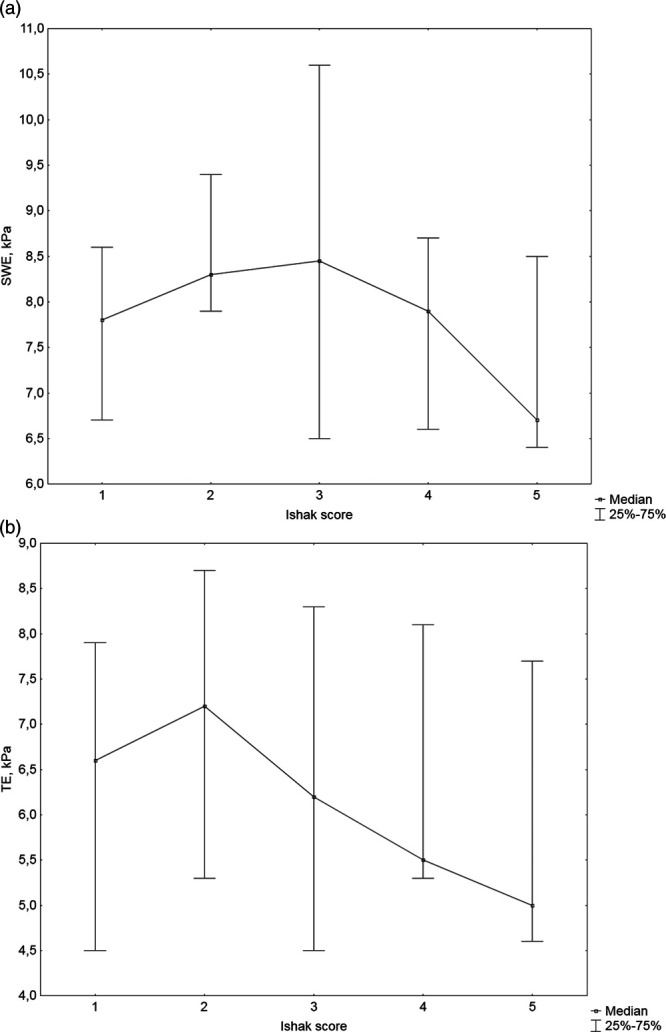
Liver stiffness measurements by Ishak score. (a) Transient elastography. (b) Shear wave elastography.

CPA percentage increased with the Ishak score, generally in a linear fashion (Fig. 1) and a significant correlation was observed (*r* = 0.45; *P* = 0.001) (Table 3). Transient elastography and SWE were highly and significantly correlated (*r* = 0.80; *P* = 0.0001) (Table 3). When liver stiffness measurements were plotted against the measured Ishak scores, it appeared that transient elastography and SWE increased in the early stages of disease up to Ishak score of 2 or 3 and then decreased in the advanced stages (Fig. 2a and b). Transient elastography and SWE did not significantly correlate with the Ishak score, CPA or HAI (Table 3); therefore, a cutoff value for liver cirrhosis could not be proposed.

**Table 3.** Correlation between patients’ clinical characteristics, Ishak score, collagen proportionate area, transient elastography and shear wave elastography measurements (using the Spearman *r* test)

Variables	*P* value			
	Ishak score	Collagen proportionate area	Transient elastography	Shear wave elastography
Sex	0.443	0.485	0.880	0.947
Age	0.910	0.998	0.718	0.702
Age at diagnosis	0.977	0.734	0.897	0.930
Wilson’s disease duration	0.872	0.683	0.573	0.744
Esophageal varices	0.173	0.476	0.693	0.601
Cirrhosis on imaging	0.356	0.104	0.630	0.172
Ishak score	–	**0.001** ^a^	0.668	0.496
Steatosis	0.543	0.270	0.232	0.627
Histology activity index	0.102	0.090	0.700	0.285
Mean collagen proportionate area	**0.001** ^a^	–	0.567	0.230
Transient elastography	0.668	0.567	–	**0.0001** ^b^
Shear wave elastography	0.496	0.230	**0.0001** ^b^	–
AST	0.243	0.729	0.887	0.404
ALT	0.238	0.794	0.444	0.774
GGT	0.356	0.429	0.114	0.070
ALP	0.538	0.427	0.670	0.826
Total bilirubin	0.399	0.374	0.442	0.156
Creatinine	0.456	0.940	0.871	0.588
Albumin	0.251	0.969	0.776	0.402
INR	0.888	0.864	0.666	0.096
Platelets	0.328	0.619	0.310	0.878
De Ritis ratio	0.807	0.978	0.625	0.346
FIB-4	0.836	0.699	0.505	0.227
APRI	0.188	0.606	0.848	0.490
Bonacini score	0.365	0.586	0.427	0.936
MELD	0.660	0.498	0.745	0.130

ALT, alanine aminotransferase; AST, aspartate aminotransferase; GGT, gamma-glutamyltransferase; ALP, alkaline phosphatase; APRI, AST-to-platelet ratio index; INR, international normalized ratio; MELD, model of end-stage liver disease.

a *r* = 0.45.

b *r* = 0.80.

There was no significant correlation between measurements of CPA, transient elastography or SWE and any of the clinical features and laboratory prognostic scores tested (De Ritis ratio, FIB-4, APRI, Bonacini and MELD) (Table 3).

## Discussion

In this study, we demonstrated that a digital CPA method for collagen content analysis corresponded with the Ishak score and may be useful for staging liver fibrosis in Wilson’s disease patients. Calvaruso *et al*. [17] proved that CPA correlates with Ishak score in patients with viral hepatitis C. Furthermore, Krawczyk *et al*. [18] showed that CPA correlates with liver fibrosis in histological Laennec score in explanted liver of patients with PSC.

We did not find a linear correlation between transient elastography and SWE measurements and histological grading of liver fibrosis. Karlas *et al*. [19] evaluated the feasibility and diagnostic accuracy of transient elastography and serologic indices of fibrosis in 50 patients with Wilson’s disease. The value of 6.1 kPA was determined as the best cutoff measurement to discriminate cirrhosis; however, there was no histopathological verification of transient elastography findings. The staging of liver fibrosis in this study was based on laboratory results and liver morphology in imaging studies.

Sini *et al*. [20] compared results of liver stiffness evaluated by transient elastography, serum fibrosis markers and liver biopsy results in 28 young (aged 25 ± 7 years) treated patients with Wilson’s disease and the METAVIR F1-F4 scoring system was used. Transient elastography values increased proportionally with progression of the histological fibrosis stage, and the authors proposed a cutoff value of 6.6 kPA for significant fibrosis and a cutoff value 8.4 kPA for advanced fibrosis. Similar to our results, liver stiffness values were overlapping in different fibrosis categories and the transient elastography values did not differ between METAVIR F1 vs F4, F2 vs F3, F3 vs F4. There was a difference between F1 vs F3 and F1 vs F4.

In a study by Behairy *et al*. [21], transient elastography values were compared with the Ishak score in pediatric populations with autoimmune hepatitis (*n* = 50), hepatitis C (*n* = 20) or Wilson’s disease (*n* = 20). There was a correlation of transient elastography results with the Ishak score irrespective of liver diseases etiology; however, values of liver stiffness differed between liver disease etiologies. The studied Wilson’s disease population was small, so different liver fibrosis categories were not adequately represented. Six Wilson’s disease patients were classified according to the Ishak score as F2 or F3 and 14 patients were categorized in a range F4–F6.

The lack of linear correlation of transient elastography and SWE with histological assessment of liver fibrosis in Wilson’s disease may be a consequence of the varying liver involvement patterns of different stages of the disease. This variation includes microvesicular and macrovesicular steatosis, glycogenation of nuclei, mitochondrial changes, such as widened intercristal space, vacuolization and increased granularity of the matrix, arrival of the portal and periportal inflammation composed of lymphocytes and plasma cells, destruction of the limiting plate, parenchymal necrosis, bridging fibrosis, intracytoplasmic eosinophilic Mallory bodies, micronodular or mixed micro–macro nodular cirrhosis, cavitation, deposition of copper salts, lipogranulomas, etc. [22,23]. It was shown in a single-center study that liver biopsy findings did not correlate significantly with clinical parameters or initial presentation in Wilson’s disease patients [24].

Along with the various liver involvement patterns in Wilson’s disease, copper content may play a role in the discrepancy between elastography and the histological liver fibrosis assessment. Paternostro *et al*. [25] examined 55 patients with liver biopsy and repetitive transient elastography. Wilson’s disease patients were examined with transient elastography at the time of diagnosis or during regular outpatient visits during treatment. The cutoff value of 12 kPA in transient elastography correctly classified patients with cirrhosis in an untreated population (*n* = 6), but the accuracy of transient elastography was low in the treated population (*n* = 49). These results have been confirmed in another study by Paternostro *et al*. [26]. Based on the analysis of data from 188 Wilson’s disease patients, the authors concluded that transient elastography and noninvasive fibrosis scores are useful to identify cirrhosis in patients with recently diagnosed Wilson’s disease, and proposed a new cutoff value of liver stiffness for cirrhosis ≥9.9 kPA [26]. In a study by Stefanescu *et al*. [27], in nine children with Wilson’s disease, liver stiffness was the highest at the time of diagnosis and decreased during treatment in parallel with an increase in urinary copper concentration. Based on these findings, repeated liver stiffness measurements may be useful in assessing the progression/regression of liver fibrosis in Wilson’s disease.

Several studies have assessed the potential of noninvasive laboratory-based scores in predicting advancement of liver disease. Eminler *et al*. [28] demonstrated that APRI was significantly higher in patients with hepatitis B and C with high levels of fibrosis compared with those with low fibrosis. Karlas *et al*. [19] and Paternostro *et al*. [25,26] showed a correlation between transient elastography and several noninvasive scores (APRI, FIB-4 and Forns). In contrast, the present study did not prove the potential of noninvasive scores as an outpatient screening tool for the identification of treated patients with Wilson’s disease prone to liver cirrhosis.

Possible limitations of the current study are the small number of individuals involved and its retrospective nature.

In conclusion, CPA assessment may be an effective method for the assessment of liver fibrosis in patients with Wilson’s disease. Single time-point transient elastography and SWE had a limited value in the precise assessment of liver fibrosis in treated patients with Wilson’s disease; however, repeated liver stiffness measurement may be useful in assessing progression/regression.

## Acknowledgements

**Conflict of interest**

There are no conflicts of interest.
